# “Shaping” of cell signaling via AKAP-tethered PDE4D: Probing with AKAR2-AKAP5 biosensor

**DOI:** 10.1186/1750-2187-7-4

**Published:** 2012-05-14

**Authors:** Salih S Koçer, Hsien-yu Wang, Craig C Malbon

**Affiliations:** 1 Department of Pharmacological Sciences, Health Sciences Center, BST-7, SUNY at Stony Brook, School of Medicine, Stony Brook, New York 11794-8651, USA; 2 Department of Physiology and Biophysics, SUNY at Stony Brook, School of Medicine, Stony Brook, New York 11794-8661, USA

**Keywords:** AKAP5, AKAP12, AKAR2, β-adrenergic receptor, PDE4D, Protein kinase A, Scaffold, Tethered

## Abstract

**Background:**

PKA, a key regulator of cell signaling, phosphorylates a diverse and important array of target molecules and is spatially docked to members of the A-kinase Anchoring Protein (AKAP) family. AKAR2 is a biosensor which yields a FRET signal *in vivo*, when phosphorylated by PKA. AKAP5, a prominent member of the AKAP family, docks several signaling molecules including PKA, PDE4D, as well as GPCRs, and is obligate for the propagation of the activation of the mitogen-activated protein kinase cascade from GPCRs to ERK1,2.

**Results:**

Using an AKAR2-AKAP5 fusion “biosensor”, we investigated the spatial-temporal activation of AKAP5 undergoing phosphorylation by PKA in response to β-adrenergic stimulation. The pattern of PKA activation reported by AKAR2-AKAP5 is a more rapid and spatially distinct from those “sensed” by AKAR2-AKAP12. Spatial-temporal restriction of activated PKA by AKAP5 was found to “shape” the signaling response. Phosphatase PDE4D tethered to AKAP5 also later reverses within 60 s elevated intracellular cyclic AMP levels stimulated by β-adrenergic agonist. AKAP12, however, fails to attenuate the rise in cyclic AMP over this time. Fusion of the AKAP5 PDE4D-binding-domain to AKAP12 was found to accelerate a reversal of accumulation of intracellular cyclic AMP.

**Conclusion:**

AKAPs, which are scaffolds with tethered enzymes, can “shape” the temporal and spatial aspects of cell signaling.

## Introduction

Scaffold proteins that dock the regulatory subunits (i.e.*,* RI//RII) of cyclic AMP (cyclic AMP)-dependent protein kinase A (PKA, A-kinase) constitute a family of 40+ members. These so-called “A-Kinase Anchoring Proteins” (AKAPs) have been revealed as essential to cellular signaling [1]. AKAPs dock PKA and act as molecular “tool boxes” capable of targeting the protein kinases [e.g., PKA as well as protein kinase C (PKC) and the Src-family of tyrosine kinases], phosphoprotein phosphatases (such as protein phosphatase-2B), and adaptor molecules to microdomains in cells [2-4]. AKAPs likely nucleate supermolecular signaling complexes that facilitate productive interactions among protein kinases, phosphoprotein phosphatases, phosphodiesterases (PDE), ion channels, adaptor molecules (like Grb2), and G protein-coupled receptors (GPCR) [5]. Two AKAPs, AKAP5 (also known as AKAP75/79) and AKAP12 (also known as gravin, AKAP 250, and SSECKS) dock to β-adrenergic receptors [2,5-9]. Human epidermoid carcinoma A431 and human embryonic kidney cells (HEK) 293 cells express several AKAPs, including AKAP5 and AKAP12, and a full complement of the prototypic GPCR, notably the β_2_-adrenergic receptor [10-13].

Detailing the spatial-temporal dynamics of molecules that actively participate in a signaling pathway during signaling has been a challenge [14]. Immunohistochemical and imaging of auto fluorescently-tagged molecules cannot distinguish between molecules actively participating in signaling versus the *entire* cellular complement of the molecule. Study of the subset of these molecules actively involved in the signaling in live cells is especially challenging, as they constitute a vanishing small percentage of the full cellular complement. “*Biosensors*” capable of acting as reporters in real time for activation of a protein kinase (e.g., AKAR/AKAR2 reporters of PKA activation) offer a powerful, novel strategy targeting spatial-temporal features of signaling catalyzed by PKA [15]. AKAR2 is a biosensor for PKA-catalyzed phosphorylation, composed of a phosphoamino acid-binding sequence of the Forkhead Homology domain and a citrine, a less pH-sensitive variant of enhanced yellow fluorescent protein (YFP) [14,15]. PKA-catalyzed phosphorylation of AKAR2 consensus sequence provokes binding of the phosphoamino acid-binding domain, closing the proximity of CFP and YFP, provoking fluorescence energy transfer (FRET) that can be measured and imaged in real time in live cell [14,15].

We have sought a hybrid approach that combines the power of the AKAR2 biosensor with a desire to measure events catalyzed by PKA, confined intentionally to single AKAPs, which are PKA substrates [16-19]. PKA catalyzed phosphorylation of AKAP5 is central to its function, mediating the mitogen-activated protein kinase cascade. Phosphorylation of AKAP12 is essential to the ability of AKAP12 to dephosphorylate, resensitize and recycle GPCRs [18]. PKA-catalyzed phosphorylation per se regulates AKAP scaffolds, including those that dock ion channels and/or GPCRs [1,6,8,16]. Earlier we reported the engineering and use of the first AKAP biosensor, AKAR2-AKAP12 [19]. Herein, a novel fusion protein of the biosensor AKAR2 with the AKAP5 is created and characterized. AKAP5 is organized similarly to AKAP12, but is only one-quarter of the size of AKAP12. This novel fusion protein retains all the functional capability of the native AKAP5 and successfully acts as a biosensor. Activation of AKAR2-AKAP5 in live cells undergoing β-adrenergic activation of mitogen-activated protein kinase (MAPK) cascade can be monitored in live cells. We explore the impact of tethered PKA and tethered PDE4D on the “shape” of the cellular responses mediated by AKAP5, using AKAP12 to test findings obtained with the smaller AKAP.

## Results

### Construction, expression, and function of AKAR2-AKAP5 fusion biosensor

**Figure 1:**
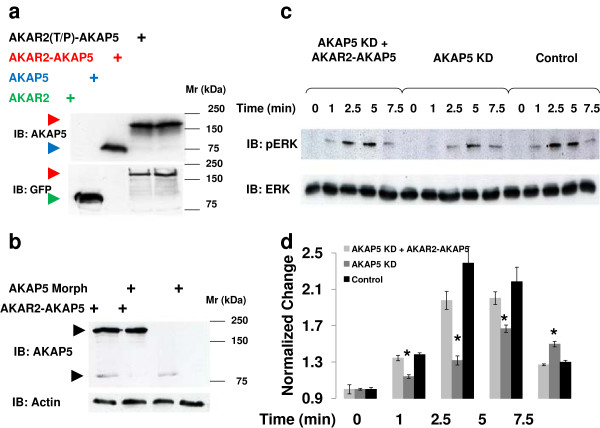
**Expression and functional equivalence of AKAR2-AKAP5 fusion protein to native AKAP5.** HEK293 cells were transfected with an expression vector harboring one of the following: either AKAR2; AKAP5; AKAR2-AKAP5 or AKAR2(T/P)-AKAP5. Cell lysates were analyzed by SDS-PAGE and resolved proteins transferred to nitrocellulose membranes. Blots were probed with antibodies specific for AKAP5 or GFP (**A**). HEK 293 cells were transfected with antisense morpholino oligonucleotides (AKAP5 morph) targeting the 5′ untranslated region (UTR) of human AKAP5. At 24 h later, AKAP5-deficient cells were divided into two batches: one was transfected with empty plasmid; and the other transfected with plasmid encoding AKAR2-AKAP5 fusion protein. AKAP5-deficient cells and wild-type cells each were stimulated with 10 μM isoproterenol (ISO). Activation of the mitogen-activated kinases ERK1,2 was established by activation- and phospho-specific antibody staining of immunoblots. Blots were probed with antibodies specific for either activated- and phosphorylated- pERK or pan-ERK (**C**). The efficiency of the antisense morpholinos used to suppress endogenous AKAP5 in these experiments is shown (**B**). β-actin is used as internal control for loading equivalence and was stained with anti-actin antibodies. The changes in ISO-stimulated activation of ERK in celles in which AKAP5 was knocked-down (KD) and then rescued by exogenous expression of AKAR2-AKAP5 as displayed in *Panel C* are plotted (**D**). The results displayed are mean values and s.e.m. derived from at least three separate experiments. *, denotes significance of *p* < 0.05.

AKAP5 functions in regulation of L-type Cav1.2 currents and the activation of the mitogen-activate protein kinases ERK1,2 [8,20,21]. Landmarks in the topology of AKAP5 are an N-terminal region (which unlike AKAP12 is not N-myristoylated) that displays three positively-charged domains (PCDs) which target the scaffold to the inner leaflet of the cell membrane via electrostatic interactions with phospholipids, a central region that interacts with the GPCR β_2_-adrenergic receptor, and a C-terminal region to which the RII subunit of PKA docks. We fused AKAR2, which biosenses PKA-catalyzed phosphorylations *in vivo*, to the N-terminus of AKAP5. The fusion product is AKAR2-AKAP5 was probed for biological activity. AKAP5 fused with a threonine-to-proline substituted, inactive version AKAR2(T/P) was constructed as a negative control [AKAR2(T/P)AKAP5], and both constructs were expressed in human embryonic kidney (HEK) 293 cells (Figure 1). Cells were transfected with an expression vector harboring a single form, AKAR2-AKAP5, AKAR2(T/P)-AKAP5), AKAR2, and native AKAP5. Cells were harvested, lysed, and samples of lysates subjected to SDS-polyacrylamide gel electrophoresis and to immunoblotting (Figure 1A). Staining of the resolved proteins by specific antibodies revealed AKAR2 (*M*_*r*_ = 80 kDa via anti-GFP antibodies), AKAP5 (*M*_*r*_ = 79 kDa via anti-AKAP5 antibodies). Both AKAP5-derived AKAR2 fusion proteins displayed *M*_*r*_ = 170 kDa in immunoblots stained with anti-AKAP5 antibodies.

Knock-down (KD) of AKAP5 with antisense morpholinos targeting the 5′-UTR of the human AKAP5 suppressed expression of endogenous AKAP5 (Figure 1B). Expression of the AKAR2-AKAP5 fusion biosensor, in contrast, was not targeted by the morpholinos (Figure 1B). KD of endogenous AKAP5 attenuated the activation of ERK1,2 in response to stimulation with the β-adrenergic agonist isoproterenol [ISO, 10 μM](Figure 1C, D). In control cells (i.e., untreated with morpholinos antisense to AKAP5), ISO stimulated a rapid, ~2.0-fold increase in activated, phosphorylated ERK1,2. The response was quantified in immunoblots stained with activation- and phospho-specific antibodies to ERK1,2. The cellular content of ERK1,2 was assayed by immunoblotting and staining with anti-ERK1,2 antibodies (bottom, Figure 1C). Expression of AKAR2-AKAP5 in the AKAP5-deficient cells fully rescued of the beta-adrenergic activation of ERK1,2 (Figure 1D). The time-course of β-adrenergic-induced ERK1,2 activation was rapid (observed within 1 min of addition of ISO), peaked within 2–3 min of β-adrenergic agonist, and was returned to baseline within ~10 min. Thus, AKAR2-AKAP5 is readily expressed and fully functional with respect to activation of ERK1,2 by β-adrenergic agonist.

### AKAP5 and AKAP12 differentially localize

**Figure 2:**
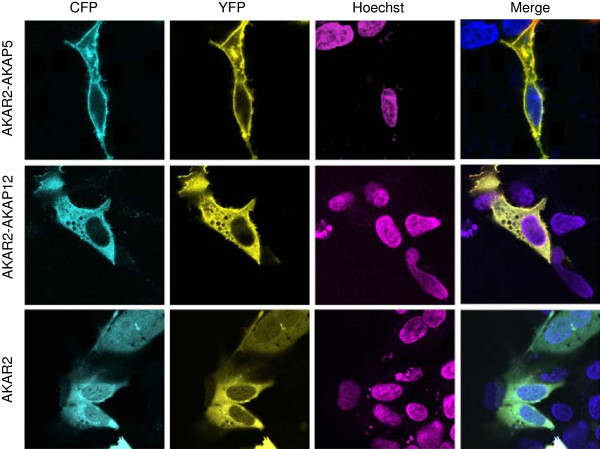
**Spatial localization of AKAR2-AKAP5 and AKAR2-AKAP12 in resting cells.** Fluorescence images of AKAR2, AKAR2-AKAP5, AKAR2-AKAP12 and were recorded at 473-495 nm for CFP and 527-591 nm for YFP, in HEK 293 cells. The nuclei were stained with Hoechst 33258. Individual panels display the signal from CFP only, YFP only, or Hoechst only. The merged image of all three inputs is provided. Experiments were repeated at least three times with equivalent results.

In resting cells, absent any stimulation with β-adrenergic agonist, AKAR2-AKAP5 localizes to the cell membrane [22-25]. Three PCDs tightly bind native AKAP5 to the negatively-charged, inner cell membrane [26,27]. AKAR2-AKAP5 likewise localizes to the cell membrane and some in the nucleus (Figure 2), the presence of the AKAR2 moiety does not disrupt this pattern of localization. In resting cells, AKAP12 can be found in the cytosol and enriched at the plasma membrane [19-21]. AKAR2-AKAP12 localizes in the same fashion as native AKAP12 (Figure 2), also displaying three PCDs that are essential for function [6,16,17]. The localization of AKAR2 was explored in these same HEK293 cells (Figure 2). AKAR2 is uniformly distributed through the cytosol, as noted previously [15]. Fluorescence images were recorded at 473–495 nm targeting the CFP moiety and 527–591 nm for the YFP moiety. Nuclei, stained with Hoechst 33258 dye, were made visible at 325 nm (Figure 2). In the “merge” lane, recorded images are displayed for CFP, for YFP, and for Hoechst 33258 dye.

### *In vivo* biosensing of PKA-catalyzed phosphorylation of AKAPs

**Figure 3:**
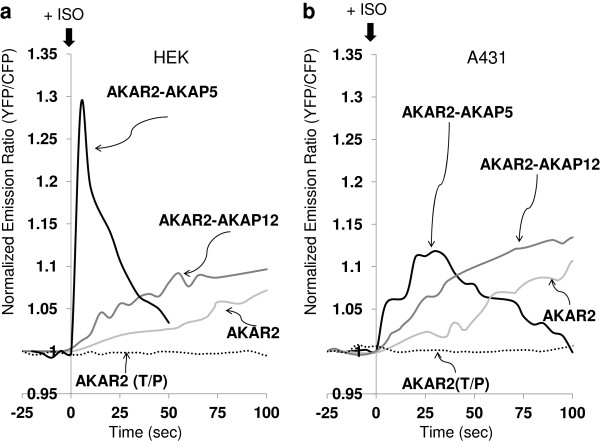
**Continuous *in vivo *imaging of AKAP5 and AKAP12 phosphorylation by PKA following stimulation with β-adrenergic agonist, as reported by their AKAR2 biosensor derivatives.** HEK 293 (**A**) and A431 cells (**B**) were transfected with the expression vectors for AKAP5 and AKAP12 biosensors, as well as the AKAR2 sensor itself. At 24 h post transfection, cells were washed twice with DMEM with 4.5 g/L glucose and sodium pyruvate lacking L-glutamine and phenol red. Cells were treated at time = 0 with 10 μM isoproterenol (ISO). The ratios of yellow-to-cyan signals were recorded every 5 s making use of METAFLUOR software. The data are normalized by dividing all ratios by the emission ratio captured in the basal sate before the stimulation. Experiments were repeated at least three times with equivalent results.

FRET signals derived from AKAR2-AKAP5 and AKAR2-AKAP12 were continuously monitored (sampling at 5 s intervals) in HEK 293 and another model cell line human epidermoid carcinoma A431 cells (Figure 3). Cells were stimulated with isoproterenol (ISO) and FRET signals were recorded for 100 s. Treatment HEK cells with ISO provokes a rapid FRET signal from AKAR2-AKAP5 that peaked within 10 s (Figure 3A). ISO stimulated a less robust, progressive FRET signal which essentially plateaus by 60 s in HEK cells. Striking was the rapid reversal of the FRET signal derived from the phosphorylation of AKAR2-AKAP5. In HEK cells, the FRET signal derived from either AKAR2-AKAP12 or AKAR2 alone displayed no apparent reversal. These data suggest that some property of AKAP5 accounts for the rapid loss of phosphorylation in response to elevated intracellular cyclic AMP (Figure 3A).

Human epidermoid carcinoma A431 cells are a well-characterized cell line [11-13,28,29]. In A431 cells, FRET signaling of the same phosphorylation events derived from AKAR2-AKAP5 was markedly delayed in comparison to the signal derived in HEK cells (Figure 3A, 3B). The amplitude of the peak FRET signal sampled from AKAR2-AKAP5, as well as the delay in reaching and escaping from the ISO-stimulated activation were muted. FRET derived from AKAR2-AKAP12 in response to stimulation of A431 cells in response to ISO, in contrast, was similar to that observed in the HEK cells. The FRET signal derived from AKAR2 itself displayed an even slower, progressive increase in response to ISO. FRET signals from AKAR2 and AKAR2-AKAP12 showed no reversal. In cell lines expressing the inactive mutants AKAR2(T/P), AKAR2-AKAP5(T/P), and of AKAR2-AKAP12(T/P) no FRET signal was detected in response to stimulation with ISO (Figure 3A and B; data not shown).

### Fidelity of biosensing with AKAP phosphorylation and function

**Figure 4:**
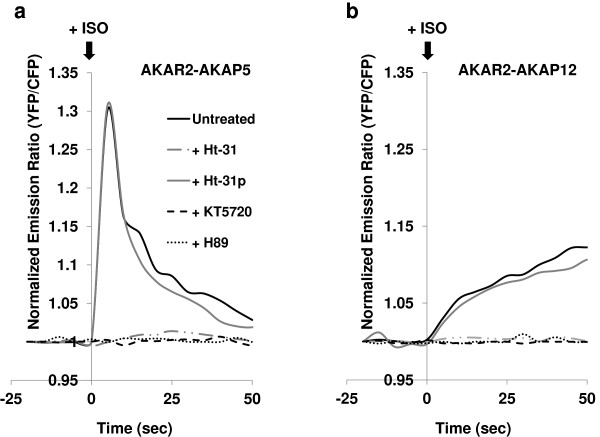
**Activation of AKAP5 *in vivo*: analysis of RII-binding site disruption and chemical inhibition of PKA.** HEK 293 cells were grown on glass bottom dishes. At 24 h later, cells were transfected with expression vectors encoding either AKAR2-AKAP5 (**A**) or AKAR2-AKAP12 (**B**). At 48 h, cells were washed twice with DMEM with 4.5 g/L glucose and sodium pyruvate lacking L-glutamine and phenol red. Cells were treated with either the sterated Ht-31 peptide (50 μM), or sterated Ht-31 control peptide (50 μM) for 30 min in a humidified atmosphere of 5% CO2 and 95% air at 37°C. For FRET data, black lines trace the biosensor signal in non-treated cells; grey lines trace the signal from Ht-31control peptide-treated cells; light grey lines trace the signal from Ht-31 peptide-treated cells. Likewise, cells expressing AKAR2-AKAP5 cells were treated with PKA inhibitors, either H89 (10 μM, dots trace the FRET signal) or KT-5720 (1 μM, dashes trace the FRET signal) (B) for 10 min before ISO stimulation. Non-treated cells represent the control. At time = -25 s the monitoring of the FRET signal commenced; at time = 0, cells were stimulated with 10 μM ISO. Experiments were repeated three times with equivalent results.

The fidelity of FRET signal as a *bona fide* reporter for the PKA-catalyzed phosphorylation of AKAP5 via the AKAR2 biosensor in cis was tested. HEK cells were challenged with two chemical inhibitors of PKA-catalyzed phosphorylation (e.g., KT5720 and H89) as well as with a small peptide (Ht-31) that blocks the ability of the PKA to dock to AKAPs (Figure 4). Cells were treated with stearated-Ht31 peptide, a cell-permanent dominant-interfering peptide that blocks the interaction between RI/II subunits of PKA and their binding site of AKAPs [30-33]. Preloading the cells with stearated-Ht31 for 30 min abolished the FRET signal expected from AKAR2-AKAP5 in response to ISO (Figure 4A). Displacement of the PKA from the AKAP5 abolished FRET signal from the biosensor in response to ISO. Preloading cells with the prolyl-substituted, stearated-Ht31p peptide, which is unable to act as a dominant-interfering peptide for AKAPs, displayed the typical spike in FRET signaling from AKAR2-AKAP5. Treating cells with H89 (i.e., 5-isoquinolinesulfonamide), a cell-permeable, potent PKA inhibitor, abolished the AKAR2-AKAP5 derived FRET signal provoked in response to ISO. Treating the cells with a semi-synthetic cell-permeable, specific and potent inhibitor of PKA (i.e., KT5720) also abolished ISO-stimulated FRET signal from the AKAP5 biosensor (Figure 4A).

HEK cells expressing the AKAR2-AKAP12 biosensor likewise were tested for fidelity with phosphorylation and function. This larger AKAP displays many of the same docking partners, including PKA, PKC, and β_2_-adrenergic receptors as its 3-fold smaller AKAP member, AKAP5. FRET signal from the two AKAP biosensors displayed the same response to chemical inhibitors of PKA and block of RI/II binding (Figure 4A, B). Inhibition of PKA activity blocked FRET signal of both biosensors in response to ISO. Treatment of cells to block PKA RI/II subunit docking to each AKAP also abolished the FRET signal in response to challenging the cells to ISO (Figure 4B).

### Imaging PKA-catalyzed activation of AKAP5

**Figure 5:**
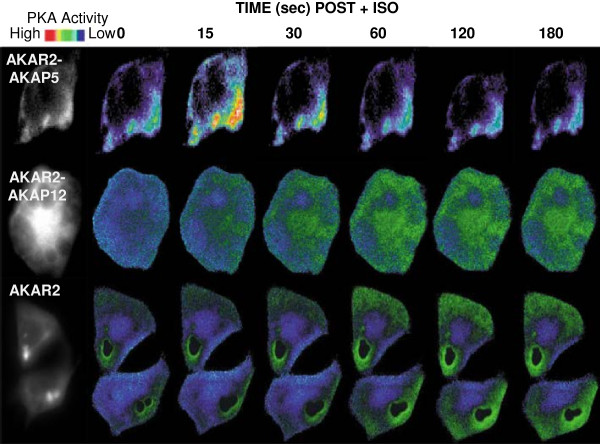
**Imaging PKA-catalyzed phosphorylation via AKAP5 versus AKAP12: response to stimulation of the cells by β-adrenergic agonist.** The YFP/CFP emission ratio images of HEK 293 cells was performed in cells expressing either AKAR2, AKAR2-AKAP5, or AKAR2-AKAP12. A time = 0, isoproterenol (ISO) was applied to the cultures. The first images displayed for each array are YFP-only images. Representative pseudocolor images display the FRET signal (PKC-catalyzed phosphorylation of the AKAR2 and two AKAP biosensors in response to ISO (10 μM). The sampling was performed at the time points indicated, recorded at every 5 s for a period of 2 min using METAFLUOR software. Experiments were repeated three times with equivalent results.

Live-cell imaging was performed during the activation of AKAR2-AKAP5 biosensor in response to β-adrenergic agonist, to ascertain the nature and pattern of PKA-catalyzed phosphorylation of this AKAP (Figure 5). The FRET signal has been “pseudo” colored, cyan constitutes low FRET and yellow constitutes high FRET. HEK cells were imaged over 180 s, “time = 0” demarcates application of ISO (Figure 5, top panel). Activation of AKAR2-AKAP5 peaks within 15 s post stimulation. By 30 s the AKAP5-derived FRET signal wanes, returning to baseline by 60 s. The FRET signal was confined to the cell membrane; essentially no signal was observed in the cytosol. The pattern of activation of AKAR2-AKAP5 was compared to that of AKAR2-AKAP12 (Figure 5, middle panel). For the entire 180 s time-course, FRET signal derived from AKAR2-AKAP12 progressively increased. The distribution of the developing FRET signal derived from AKAR2-AKAP12 occurred at the cell membrane and was propagated throughout the cytosol for AKAR2-AKAP12. AKAP5 is activated early and discretely as a pulse at the cell membrane, while AKAP12 is activated progressively without apparent attenuation commencing at the cell membrane throughout the cytosol. Activation of AKAR2 in response to ISO yield a FRET signal that was similar to that of AKAR2-AKAP12, slower and lacking the peak signal characteristic of AKAR2-AKAP5 (Figure 5, bottom panel). FRET signals from all three of the biosensors were excluded from the nucleus.

### PDE shaping of AKAP5 phosphorylation

Several members of the AKAP family dock cyclic AMP phosphodiesterases (PDE), in addition to protein kinase and phosphoprotein phosphatases. By degrading cyclic AMP, PDEs have been reported to “condition” cellular microenvironments [34,35]. PDEs particularly members of the PDE4 family play central roles in reversing the activation of substrate proteins by PKA [35-40]. AKAP5 has been reported to tether PDEs in multiple ways [7,35,41], whereas AKAP12 displays no putative PDE-binding site. To test role of PDE in AKAP5 we adopted three interrelated approaches. The first was to create a dominant interfering peptide (DIP) from the AKAP5 sequence that would sequester PDE via the tethering site, but one that would not localize to the cell membrane. The first step was to delete all three N-terminal positively-charged domains (i.e., PCD1, PCD2, and PCD3) that localize AKAP5 to the cell membrane [26,27], providing a “sink” to which PDE can tether away from the membrane. Next, a point mutation at the RII binding domain of this 146-427 AKAP5 fragment was inserted. Mutation of the RII binding site insured that the fragment would not be a “sink” for PKA. This mutated 146-427 peptide was tested to ascertain if it could function to sequester PDE away from AKAP5. HEK cells were stably transfected to express an HA-tagged version of the N-terminally-truncated, mutated AKAP5 146-427 (termed “Δ1-145,T/P395”). An expression vector harboring AKAR2-AKAP5 was transfected into either wild-type cells or cells expressing Δ1-145,T/P395. In immunoblots stained with anti-PDE4D5, cells expressing GFP-tagged AKAR2-AKAP5 display less bound PDE4D5 when co-transfected with Δ1-145,T/P395 peptide (Additional file 1: Figure S1A), i.e., the presence of the DIP reduces the amount of PDE tethered to AKAP5.

**Figure 6:**
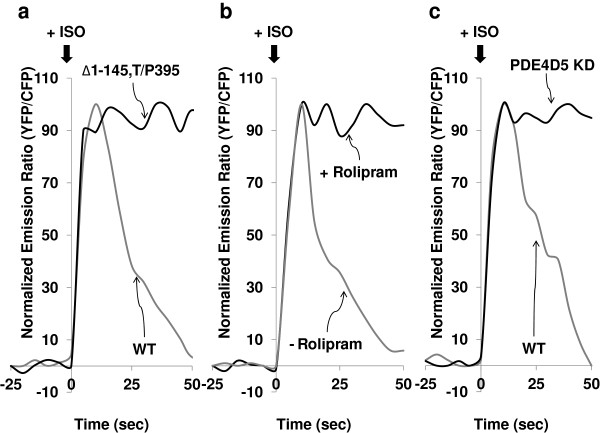
**Activation of AKAP5 is reversed by tethered PDE.** Wild-type HEK cells or cells stably transfected with Δ1-145,T/P395, an N-terminally truncated AKAP5 (lacking the PCDs that function in membrane localization) with a T/P395 mutation, were co-transfected with AKAR2-AKAP5 biosensor (**A**). At 24 h post biosensor transfection, cells were treated at time = 0 with 10 μM ISO (A,B,C). HEK293 cells were transiently transfected with AKAR2-AKAP5 and pretreated with either vehicle alone (DMSO) or 10 μM Rolipram (a chemical inhibitor of PDE4) for 10 min. These cells were treated with 10 μM ISO at time = 0 (**B**). Wild-type HEK293 and cells in which PDE4D5 was knocked down both were transiently transfected with AKAR2-AKAP5 biosensor. Cells were treated with 10 μM ISO at time = 0 (**C**). The ratios of yellow-to-cyan were recorded at every 5 s using METAFLUOR software and normalized by dividing all ratios by the emission ratio before stimulation. Experiments were repeated three times with equivalent results.

The FRET signal from AKAR2-AKAP5 was monitored in cells co-transfected to express Δ1-145,T/P395 DIP (Figure 6). In the absence of Δ1-145,T/P395 DIP, cells demonstrated a rapid, transient peak of FRET signal in response to ISO. When these cells expressed the Δ1-145,T/P395 DIP, the character of the FRET signal from PKA-catalyzed phosphorylation of AKAP5 changed dramatically (Figure 6A). Although the initial peak FRET signal from the AKAP5 biosensor was equivalent to the control in both sets of cells, cells expressing the Δ1-145,T/P395 DIP abolished sharp reversal of the FRET signal commencing at 10 s (Figure 6A). The FRET signal derived from AKAR2-AKAP5 persisted up to 50 s, with no indication of a decline in the cells expressing the Δ1-145,T/P395 DIP. These observations argue that interfering with the availability of AKAP5 to dock PDE abolished the rectification of PKA-catalyzed phosphorylation, presumed to reflect an inability to degrade the intracellular cyclic AMP in the close proximity of the AKAP5 biosensor and tethered PDE.

A second approach made use of the specific PDE4 inhibitor Rolipram (Figure 6B). Treating cells with Rolipram would block the catalytic activity of PDE, including that tethered to AKAP5. In the absence of Rolipram, ISO stimulated a rapid, transient peak of FRET signal from AKAR2-AKAP5. Inhibiting PDE activity did not alter the initial rapid peak of FRET signal, but rather blocked the reversal of the signal that rapidly follows in the absence of PDE inhibitor. Since Rolipram can inhibit several PDEs and only PDE4D5 is the member tethered to AKAP5 uniquely, we sought to ask a more precise question about the role of this PDE in AKAP5 function. Making use of siRNA specifically targeting PDE4D5, the expression of PDE4D5 was knocked-down (KD) (Figure 6C). Suppression of PDE4D5, like Rolipram, did not alter the initial peak of AKAR2-AKAP5-derived FRET signal, but effectively abolished the reversal of the signal which reflects loss of PKA-catalyzed phosphorylation of the AKAP.

**Figure 7:**
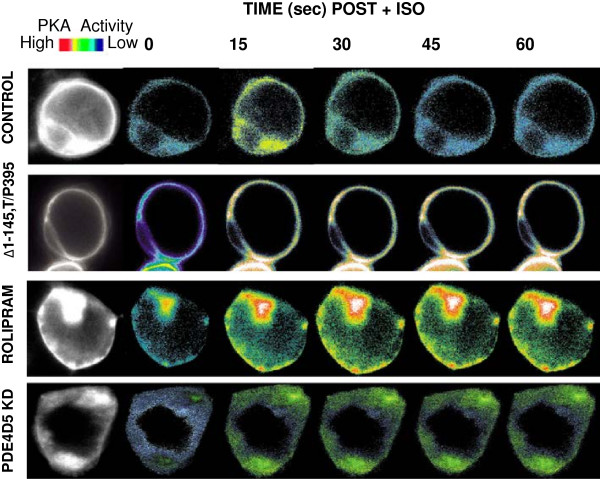
**Probing the role of tethered PDE in AKAP5-based PKA-catalyzed phosphorylation.** Wild-type HEK293 cells were untreated (Controls) or pretreated with siRNAs targeting PDE4D5, or stably transfected to express the Δ1-145,T/P395 AKAP fragment (a PDE-binding “sink”). These different cell treatments were followed by transfection with the AKAR2-AKAP5 biosensor. An addition batch of cells transfected with an expression vector harboring AKAR2-AKAP5 were pretreated with the PDE4-specific inhibitor Rolipram (10 μM for 10 min). The images display the YFP/CFP emission ratio images of HEK 293 cells derived from the AKAR2-AKAP5 biosensor in cells stimulated with ISO. The first images of each array are YFP-only images. Cells were treated with at time = 0 with 10 μM ISO and the imaging commenced. Representative pseudocolor images show the change in FRET in response to ISO (10 μM) stimulation at various time points recorded at every 5 s by METAFLUOR software.

*In vivo* imaging of AKAR2-AKAP5 was performed in parallel with the quantification of the FRET signal for the three approaches targeting the role of PDE (Figure 7). Expression of the Δ1-145,T/P395 DIP, treatment with Rolipram, and knock-down of PDE4D5 by siRNA abolished the attenuation of the AKAR2-AKAP5 FRET signal found 10 s post ISO, while not influencing the onset and peak time of the FRET signal immediately following ISO (Figure 7). Untreated control cells expressing the AKAP5 biosensor displayed the normal rapid and reversible peak of ISO-stimulated FRET signal (Figure 7).

**Figure 8:**
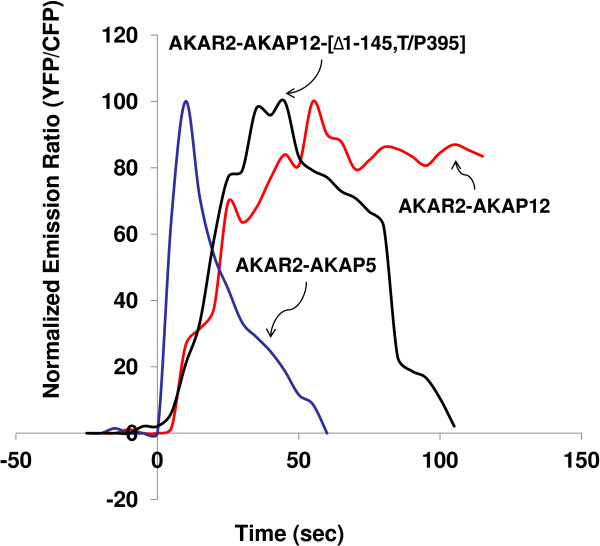
**Expression of the AKAP12 engineered with Δ1-145,T/P395 AKAP5 fragment which binds PDE effectively reverses PKA-catalyzed phosphorylation.** HEK 293 cells were placed on glass bottom dishes. At 24 h later, cells were transfected with plasmids encoding either AKAR2-AKAP5 (A), AKAR2-AKAP12 (B) or AKAR2-AKAP12-[Δ1-145,T/P395] biosensors. At 48 h, cells were washed twice with DMEM with 4.5 g/L glucose and sodium pyruvate lacking L-glutamine and phenol red. At time = 0 cells were stimulated with 10 μM ISO and the FRET signal collected and monitored in real time. The ratios of yellow-to-cyan were recorded at every 5 s using METAFLUOR software and normalized by dividing all ratios by the emission ratio before stimulation. Experiments were repeated three times with equivalent results.

An AKAP12 fusion protein was constructed that harbored the PDE4D5-binding domain of AKAP 5 (-[Δ1-145,T/P395). HEK cells were transfected with expression vectors harboring AKAR2-AKAP5, AKAR2-AKAP12, or AKAR2-AKAP12-[Δ1-145,T/P395] biosensors (Figure 8). AKAR2-AKAP5 generated a FRET signal in response to ISO that was sharp, rapid, and transient. The AKAR2-AKAP12 biosensor, in contrast, generated a FRET signal in response to ISO that was comparatively slow, lagging behind that of the AKAR2-AKAP5, and a signal that was progressive over the full time course (120 s). When the AKAR2-AKAP12-[Δ1-145,T/P395] fusion protein was expressed, which harbors the AKAP5 PDE docking site, the onset of the FRET signal in response to ISO was not unlike the AKAR2-AKAP12 (Figure 8). The AKAR2-AKAP12 sensor harboring a site to which PDE could tether now displays a sharp reversal of the activation (Figure 8). Immunoblots of pull-downs from cells expressing either AKAP12 sensor or the AKAP12 sensor harboring a PDE-binding sites reveal the association of PDE4D5 with the later, but not the former (Additional file 1: Figure S 1B). These data argue that by inserting the PDE-binding domain of AKAP5 into the AKAP12, we succeeded in shaping not the initial peak activation of the sensor, but rather the ability of AKAR2-AKAP12 to rectify the accumulation of cyclic AMP and PKA activation in its near proximity.

### AKAP5-mediated ERK1,2 activation: role of PDE

**Figure 9:**
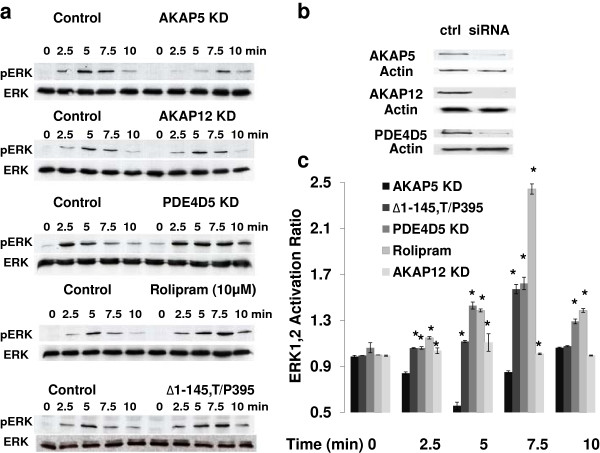
**Probing the role of PDE in AKAP5–mediated signaling to ERK1,2.** Wild-type HEK293 cells, cells treated with siRNAs targeting either AKAP5, AKAP12 or PDE4D5 or HEK293 cells stably transfected with an expression vector harboring Δ1-145,T/P395 AKAP5 fragment, or wild-type cells pretreated with the PDE4-specific inhibitor Rolipram (10 μM for 10 min) were stimulated with 10 μM ISO for different time points. Following the 10 min time course the cells were lysed and the lysates analyzed by SDS-PAGE. The resolved proteins were blotted and probed with activation- and phospho-specific antibodies to pERK1,2. Blots also were probed with antibodies to ERK1,2 (**A**). Expression of proteins targeted with siRNAs was established by immunoblotting of the same blots (**B**). Quantification of ERK1,2 activation in the three control and experimental conditions is displayed (**C**). The results shown are the activity ratio compared with the control and are the mean values and s.e.m. of three or more independent experiments. *, denotes significance of *p* < 0.05 of a ratio compare to AKAP5 deficient (AKAP5 KD) samples.

β-adrenergic agonist-driven activation of MAPK is solely mediated by AKAP5, not AKAP12 [7,8]. We investigated the role of PDE in the AKAP5-directed ERK1,2 activation (Figure 9A). Knock-down of AKAP5, AKAP12 and PDE4D5 with siRNAs suppressed expression of endogenous each targeted protein (Figure 9B). AKAP12-deficiency, as suspected, did not affect the β-adrenergic agonist-driven activation of ERK1,2. AKAP5-deficiency by siRNA suppressed ERK1,2 activation (Figure 9A and 9C). The treatment of cells with either the PDE4-specific inhibitor Rolipram, or with siRNA targeting endogenous PDE4D5 potentiated ERK1,2 activation by β-adrenergic agonist (Figure 9A and 9C), providing support for the notion that PDE4D5 functions in AKAP5 biology [7]. Furthermore, only AKAP12-deficiency by siRNA, as expected, affected β-adrenergic receptor recycling (Additional file 2: Figure S2).

## Discussion

Biosensors can offer unparalleled insights into the temporal and spatial features of signaling in live cells. The AKAR2 biosensor enables the detection in real time of PKA-catalyzed phosphorylation events in live cells [15,42,43]. We successfully engineered AKAR2 to the N-terminus of AKAP5, providing a biosensor that reports from the local environment, in *cis* orientation with respect to AKAP5. The FRET-based signal generated from the AKAP5 biosensor displayed a rapid activation in response to beta-adrenergic stimulation. Within less than 10 s the FRET signal, reflecting *in situ* PKA-catalyzed phosphorylation of AKAP5, reached peak amplitude. PKA-catalyzed phosphorylation of AKAP5 underwent rapid reversal. PCDs confine AKAP5 to the cell membrane, in close proximity to the membrane-embedded adenylylcyclase that generates cyclic AMP. The localization of AKAP5 was highly uniform, decorating the inner leaflet of the cell membrane. AKAP12, in contrast, is sequestered in close proximity to the cell membrane, but after activation migrates away from the membrane with the GPRCs undergoing resensitization and recycling catalyzed by AKAP12 [16].

The role of one particular docking partner, PDE, was of keen interest. Recent studies have revealed that the microenvironment of cyclic AMP signaling are shaped by PDEs that degrade the cyclic nucleotide [7,35,44,45]. We made use of a dominant interfering peptide fragment of AKAP5 (Δ1-145,T/P395) to sequester cellular PDE. Expression of the Δ1-145,T/P395 DIP did not alter the rapid peak of FRET signal derived from AKAP5 biosensor in response to ISO, but rather abolished the rapid reversal and transient character of the AKAP5 response. Knocking down of PDE4D5 and chemical inhibition of PDE4 also abolished the rectification of the PKA-catalyzed phosphorylation of AKAP5. Unlike AKAP5, AKAP12 does not display a binding site for PDE. PDE tethered through β-arrestin has been proposed as an alternative for AKAP12 biology [46]. When tethered to AKAP12 via the insertion of the AKAP5 PDE binding site, PDE4D5 is shown to accelerate deactivation, shaping the reversal much like that of AKAP5 (Figure 8).

**Figure 10:**
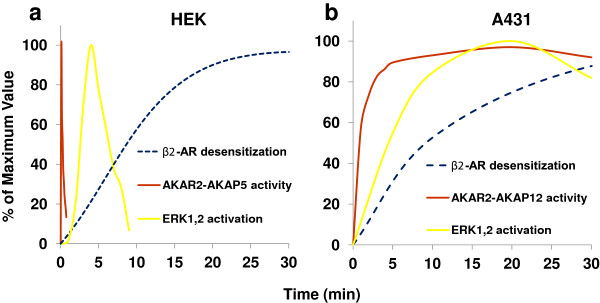
**Time-courses of activation of AKAP biosensor, ERK1,2, and of beta-adrenergic receptor desensitization: differential responses of AKAP5 versus AKAP12.** HEK 293 cells were stimulated with 10 μM ISO and sampled at times up to 30 min. HEK (**A**) or A431 (**B**) cells were sampled for three read-outs: ERK1,2 activation, AKAR2-AKAP biosensor FRET signaling, and beta-adrenergic receptor-mediated desensitization. In HEK cells, AKAR2-AKAP5 activation, ERK1,2 activation and receptor desensitization are displayed in Panel A. In A431 cells, AKAR2-AKAP12 activation, ERK1,2 activation and receptor desensitization are displayed in Panel B. “0% desensitization” denotes the magnitude of the cyclic AMP response to a second challenge with ISO compared to the first challenge. Desensitization data are plotted from earlier time-courses [47,48].

How do patterns of activation of PKA tethered to either AKAP5 or AKAP12 compare to the time-courses of ERK1,2 activation and of β_2_-adrenergic receptor desensitization? AKAP12 deficiency does not affect the β-adrenergic agonist-driven activation of ERK1,2, whereas AKAP5 deficiency does (Figure 9A and 9C). AKAP5 deficiency does not affect β_2_-adrenergic receptor desensitization (Additional file 2: Figure S2 and [8]). Activation of AKAP5 biosensor is rapid and reverses equally rapidly. Following the spike in biosensor activation is an activation of ERK1,2 mediated by AKAP5 observed within 2 min, peaks within 5 min. Desensitization of the β_2_-adrenergic receptors lags well behind the PKA-catalyzed phosphorylation of AKAP5, as well as the activation and subsequent deactivation of ERK1,2 in response to ISO. The β_2_-Adrenergic Receptor desensitization itself is half-maximal only by the time PKA and ERK1,2 activation have returned to baseline. Recycling of the internalized receptor and its resensitization is mediated by AKAP12, a response that occurs 30–60 min after agonist-induced desensitization (Figure 10). Thus, the character of the responses mediated ostensibly by PKA and propagated downstream to either ERK1,2 or receptor recycling are impacted by the AKAP shaping each response. The similarities in structural landmarks (PCDs, RII-binding sites, RBDs) and organization are obvious, but their localization and the content of the tool box mobilized by these scaffolds are the key to the eventual “shape” of the response.

## Materials and methods

### Materials

Phusion® High-Fidelity DNA polymerase was purchased from New England Biolabs (Ipswich, MA). All DNA restriction enzymes employed in this study were purchased from New England Biolabs (Ipswich, MA). Antibodies AKAP5 (cat. #: sc-17772), β-Actin (cat. # sc-47778) and GFP (cat. #: sc-8334) were purchased from Santa Cruz (Santa Cruz, CA); HA (cat. #: 11867431001) was purchased from Roche (Indianapolis; IN); and anti-PDE4D5 antibodies (cat. #: GTX14626) were obtained from GeneTex (Irvine, CA). DMEM with 4.5 g/L glucose, sodium pyruvate without L-glutamine, and phenol red were purchased from Cellgro (Manassas, VA). InCELLect™ AKAP St-Ht31 Inhibitor Peptide and St-Ht31P Control Peptide were purchased from Promega (Madison, WI). The PKA inhibitors H89, KT5720, and the β-adrenergic agonist ISO were obtained from Sigma-Aldrich, Inc (St. Louis, MO). DNA ligase, transfection reagent Lipofectamine® and all siRNAs employed in these studies was purchased from Invitrogen (Carlsbad, CA). The 35 mm glass bottom dishes with 13 mm wells were obtained from Cell E&G LLC (Huston, TX). The Nikon Eclipse TE300 microscope and a cool charge-coupled device digital camera ORCA-ER were purchased from Hamamatsu (Bridgewater, NJ). METAFLUOR 7.2 software was from Olympus (Center Valley, PA). The filter changer Lambda 10-3 and the Lambda DG-4 were purchased from Shutter Instrument (Novato, CA). Antisense morpholino oligonucleotides (morpholinos) were designed, synthesized, and purified by Gene Tools, LLC (Philomath, OR).

#### Cell culture

Human epidermoid carcinoma cells (A431) and human embryonic kidney (HEK) 293 cells were maintained as described elsewhere [6,16-19].

#### Construction of plasmids

Construction of AKAR2-AKAP12 and AKAR2(T391A)-AKAP12 plasmids was described earlier [19]. AKAR2 was engineered into the N-terminus of human AKAP12. Cellular targeting and the biological activity of the AKAP12 were unaffected by attachment of the AKAR2 moiety [19]. The cellular distribution of AKAR2-fused AKAP12 (Figure 2) and that of the endogenous AKAP12 as well as the GFP-tagged AKAP12 are identical to earlier published studies [2,6,16,19].

AKAR2-AKAP5 was constructed and engineered into an expression plasmid using the same strategy. AKAR2 sequence was amplified using PCR with primers containing HindIII and EcoRI at N- or C- termini, respectively. AKAP5 sequence was amplified with primers containing EcoRI and NotI found at N- or C- termini, respectively by using Phusion® High-Fidelity DNA polymerase (New England Biolabs; Ipswich, MA). PCR products were subcloned simultaneously into pcDNA 3.1+ vector, which then was digested with HindIII and NotI. To construct mutant AKAR2(T/P)-AKAP5 the Threonine-to-Proline substitution mutant of AKAR2 [which was generated before [19]] was amplified, digested, and subcloned into pcDNA3.1+ vector in tandem with the AKAP5 sequence. The targeting and biological activity of AKAP5 was unaffected by fusion with AKAR2 biosensor (Figure 1). The cellular distribution of AKAR2-fused AKAP5, endogenous AKAP5, and GFP-fused AKAP5 in the current studies are almost identical to those reported earlier by others [22,24,25,49].

AKAR2-AKAP12-[Δ1-145,T/P395] was created by fusing AKAP5 which is harboring the point mutation at PKA binding site and has been truncated (i.e., the first 145 amino acids deleted) making use of NotI-XhoI restriction sites in the DNA.

#### Immunoblotting

Cells were transfected with an expression vector encoding one of the following: AKAR2; AKAP5; AKAR2-AKAP5 or AKAR2(T/P)-AKAP5. Samples isolated from whole-cell lysates of the transfected cells were separated by SDS-PAGE and transferred to nitrocellulose membranes. Blots were probed with polyclonal antibodies specific for AKAP5 (1/1000 dilution), HA (1/1000 dilution) and GFP (1/1000 dilution). Western blotting technique used in this study is described elsewhere [50,51]. Antisense morpholino oligonucleotides (morpholinos) specified to the 5′ untranslated region (UTR) of human AKAP5 were employed *only* in the experiments whose results are displayed in Figure 1. The expression of AKAP5 was suppressed in HEK 293 cells by use of antisense morpholinos which targeted the 5′ untranslated region of human AKAP5, as described earlier [8]. In all other cases, knock-down of protein expression was accomplished by use of siRNAs. Where employed, antisense morpholinos were designed, synthesized, and purified by Gene Tools. At 24 h later these morpholinos-treated cells were divided into two batches, one group was transfected with empty plasmid and the other with plasmid encoding AKAR2-AKP5 fusion protein. At 72 h after initial morpholino transfection AKAP5-deficient and wild-type cells were stimulated with 10 μM ISO. Samples obtained from whole-cell lysates were subsequently subjected to SDS-PAGE and the resolved proteins transferred to nitrocellulose membranes. Blots were probed with antibodies specific for either pERK or pan-ERK. The efficiency of the antisense morpholinos in suppressing AKAP5 was complete and established by immunoblotting with anti-AKAP5 antibody (Santa Cruz; Santa Cruz, CA). β-actin was stained with antibodies (1/1000) is used as an internal control for sample loading in immunoblotting. Representative immunoblots displayed show the efficiency of suppression of the target proteins using either antisense morpholinos or siRNAs (Figure 1B and 9B).

#### Pulldowns

Pulldowns were conducted as described earlier [52,53].

#### Confocal fluorescence microscopy

Cells were plated on 35 mm glass bottom dishes with 1 3 mm well produced by Cell E&G LLC. At 24 h later, cells were transfected with plasmids encoding one of the following: AKAR2, AKAR2(T/P), AKAR2-AKAP5, AKAR2(T/P)-AKAP5, AKAR2-AKAP12 or AKAR2(T/P)-AKAP12. At 24 h later, cells were washed twice with DMEM supplemented with 4.5 g/L glucose, sodium pyruvate free of L-glutamine and phenol red (Cellgro, Manassas, VA). Cells were stained with Hoechst 33258 (diluted 1:5000 from stock solution of 10 mg/ml) for 30 min in a humidified atmosphere of 5% CO2 and 95% air maintained at 37°C. Microscopy was performed on a Zeiss LSM 510 scanning laser confocal microscope, as described [19].

#### Live cell imaging

Live cell imaging was performed as described [54]. Fluorescence ratio imaging was performed on a Nikon Eclipse TE300 microscope and a cool charge-coupled device digital camera ORCA-ER controlled by METAFLUOR 7.2 software (Olympus, Center Valley, PA). Emission ratios were obtained by using a 420DF20 excitation filter, a 450DRLP dichroic mirror, and two emission filters (475DF40 for cyan and 535DF25 for citrine) controlled by filter changer Lambda 10-3 (Shutter Instrument, Novato, CA). The Lambda DG-4 (Shutter Instrument, Novato, CA) illumination system was employed as the light source. Cells were transfected with the indicated plasmids and maintained as described above. At 24 h post transfection, cells were washed twice with DMEM with 4.5 g/L glucose and sodium pyruvate without L-glutamine and phenol red. Cells were treated at time 0 with 10 μM ISO. The ratios of yellow-to-cyan were recorded at every 5 s by METAFLUOR software and normalized by dividing all ratios by the emission ratio before stimulation. Similarly, pseudocolor images which show the change in FRET in response to ISO (10 μM) stimulation at various time points recorded at every 5 s by METAFLUOR software.

#### Recycling of β_2_-Adrenergic Receptor

A431 cells were stable transfected with Δ1-145,T/P395. AKAP12, AKAP5 or PDE4D5 were suppressed, where indicated, by specific siRNAs. The desensitization, internalization, resensitization/recycling assays were performed as described elsewhere [55].

## Abbreviations

AKAP: A-kinase anchoring protein; AKAP5: AKAP79; AKAP12: gravin, AKAP250; SSECKS: A-kinase; PKA: Protein kinase A; CFP: Cyan fluorescent protein; DIP: Dominant interfering peptide; GFP: Green fluorescent protein; GPCR: g protein-coupled receptor; HEK: Human embryonic kidney cells; MAPK: Mitogen-activated protein kinase; ISO: Isoproterenol; PDE: Cyclic AMP phosphodiesterase; SDS-PAGE: Sodium dodecyl sulfate-polyacrylamide gel electrophoresis; YFP: Yellow fluorescent protein (in these studies the variant citrine); Δ1-145,T/P395: AKAP5 146-427, dominant interfering peptide which does not bind the cell membrane, can dock PDE but not PKA.

## Competing interests

The authors declare that they have no competing interests.

## Authors’ contributions

SSK designed and constructed the AKAR2-AKAP5 biosensor, performed the experiments, gathered the data, and drafted/edited the final manuscript. HYW and CMM participated in designing of the experiments and drafted/edited the final manuscript. All authors read and approved the final manuscript.

## Supplementary Material

Additional file 1 **Figure S1.** Δ1-145,T/P395 dominant interfering AKAP5 peptide provides a PDE4D5-binding domain. (A) Wild-type HEK293 cells or HEK293 cells stably transfected with Δ1-145,T/P395 DIP were then co-transfected with the biosensor AKAR2-AKAP5. Cells were lysed and samples were subjected to pull-downs with anti-GFP antibody targeting the biosensor. The pull-downs were subjected to SDS-PAGE and immunoblotting. Blots of the resolved proteins were probed with antibodies specific against either PDE4D5 or GFP (Even though the AKAR2 does not have GFP tag, GFP antibody works against CFP and YFP). The amount of cellular PDE4D5 present in the samples was established, noted as “input.” (B) Wild-type HEK293 cells were transfected with expression vectors harboring either AKAR2-AKAP12 or AKAR2-AKAP12-[Δ1-145,T/P395]. Whole-cell lysates subjected to SDS-PAGE and immunoblotting. Blots of the resolved proteins were probed with antibodies specific against either PDE4D5 or GFP. Cellular PDE4D5 levels in the samples were stained as loading controls. Experiments were repeated at least three times with equivalent results. Click here for file

Additional file 2 **Figure S2.** Effects of targeted loss of AKAP5, AKAP12, and PDE4D5 on the resensitization/recycling of beta-adrenergic receptors. Experiments were performed with either wild-type A431 cells or cells stably transfected with an expression vector harboring the Δ1-145,T/P395 DIP of AKAP5. Cells were treated with siRNAs to knock-down (KD) AKAP12, AKAP5 or PDE4D5. These cells, deficient in AKAP5/12 or PDE4D5 then were treated with 10 μM ISO for 30 min to provoke full beta-adrenergic receptor desensitization and internalization (by 30 min). Cells then were washed free of agonist and incubated for additional 60 min to permit recovery. AKAP-mediated receptor resensitization/recycling, as measured directly using a membrane-impermanent radioligand that binds to only the complement of cell surface receptors (i.e., those receptors that have resensitized/recycled back to the cell membrane) was assayed to ascertain the impact of the expression of Δ1-145,T/P395 DIP of AKAP5 versus the loss of AKAP5, AKAP12, or PDE4D5. Cells not treated with ISO provided the control. The data shown are mean values plus/minus s.e.m. derived from at least three independent experiments. *, denotes significance of *p* < 0.05 from control cells at each time/condition. Click here for file
