# Supplementary figures and images for: “Shaping” of cell signaling via AKAP-tethered PDE4D: Probing with AKAR2-AKAP5 biosensor
Source: J Mol Signal. 2012 May 14;7:4. doi: 10.1186/1750-2187-7-4 (PMC3493269; doi:10.1186/1750-2187-7-4)

## Supplementary Figure 1

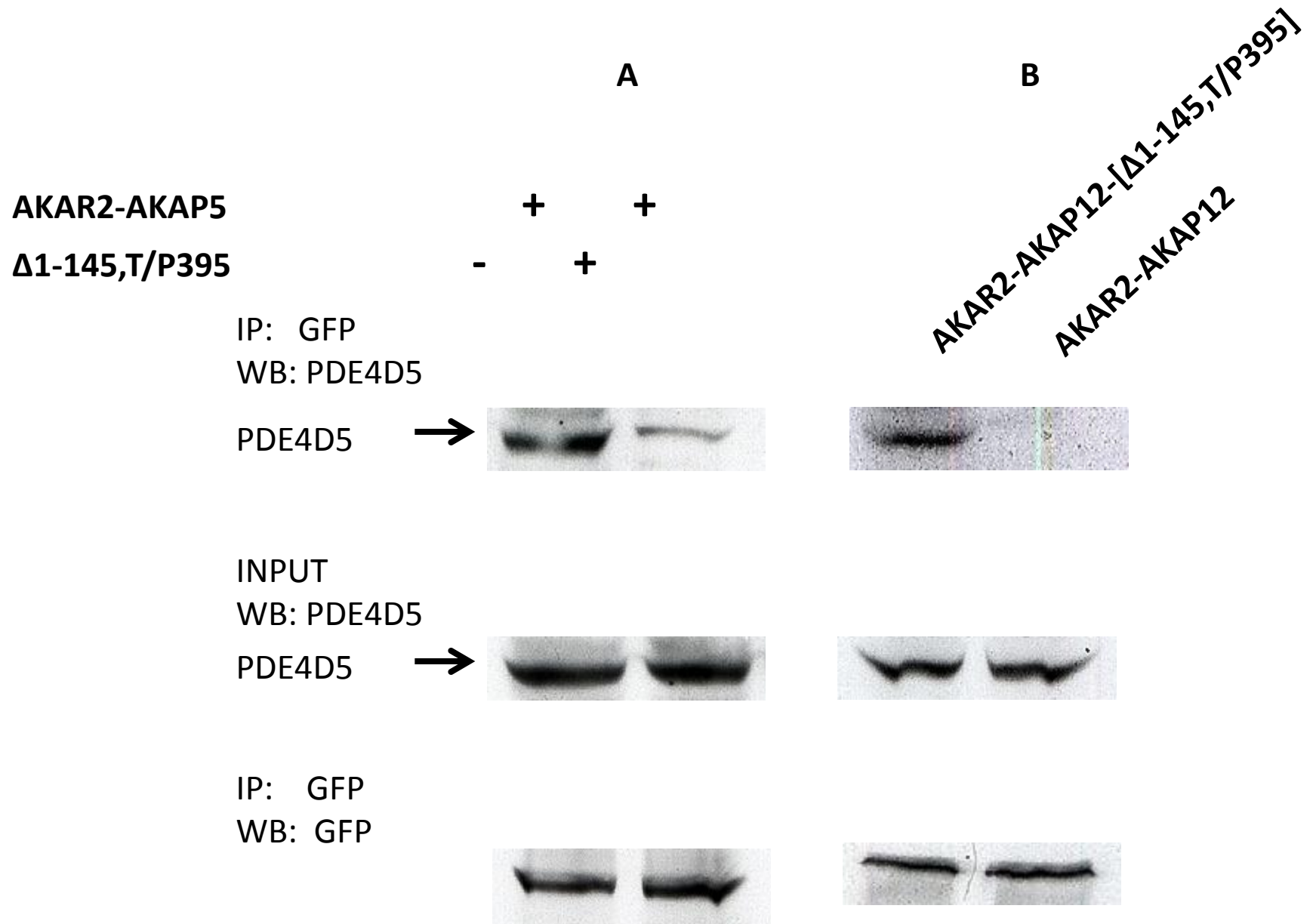

Supplement: Additional file 1 — Figure S1. Δ1-145,T/P395 dominant interfering AKAP5 peptide provides a PDE4D5-binding domain. (A) Wild-type HEK293 cells or HEK293 cells stably transfected with Δ1-145,T/P395 DIP were then co-transfected with the biosensor AKAR2-AKAP5. Cells were lysed and samples were subjected to pull-downs with anti-GFP antibody targeting the biosensor. The pull-downs were subjected to SDS-PAGE and immunoblotting. Blots of the resolved proteins were probed with antibodies specific against either PDE4D5 or GFP (Even though the AKAR2 does not have GFP tag, GFP antibody works against CFP and YFP). The amount of cellular PDE4D5 present in the samples was established, noted as “input.” (B) Wild-type HEK293 cells were transfected with expression vectors harboring either AKAR2-AKAP12 or AKAR2-AKAP12-[Δ1-145,T/P395]. Whole-cell lysates subjected to SDS-PAGE and immunoblotting. Blots of the resolved proteins were probed with antibodies specific against either PDE4D5 or GFP. Cellular PDE4D5 levels in the samples were stained as loading controls. Experiments were repeated at least three times with equivalent results. [file 1750-2187-7-4-S1.pdf]

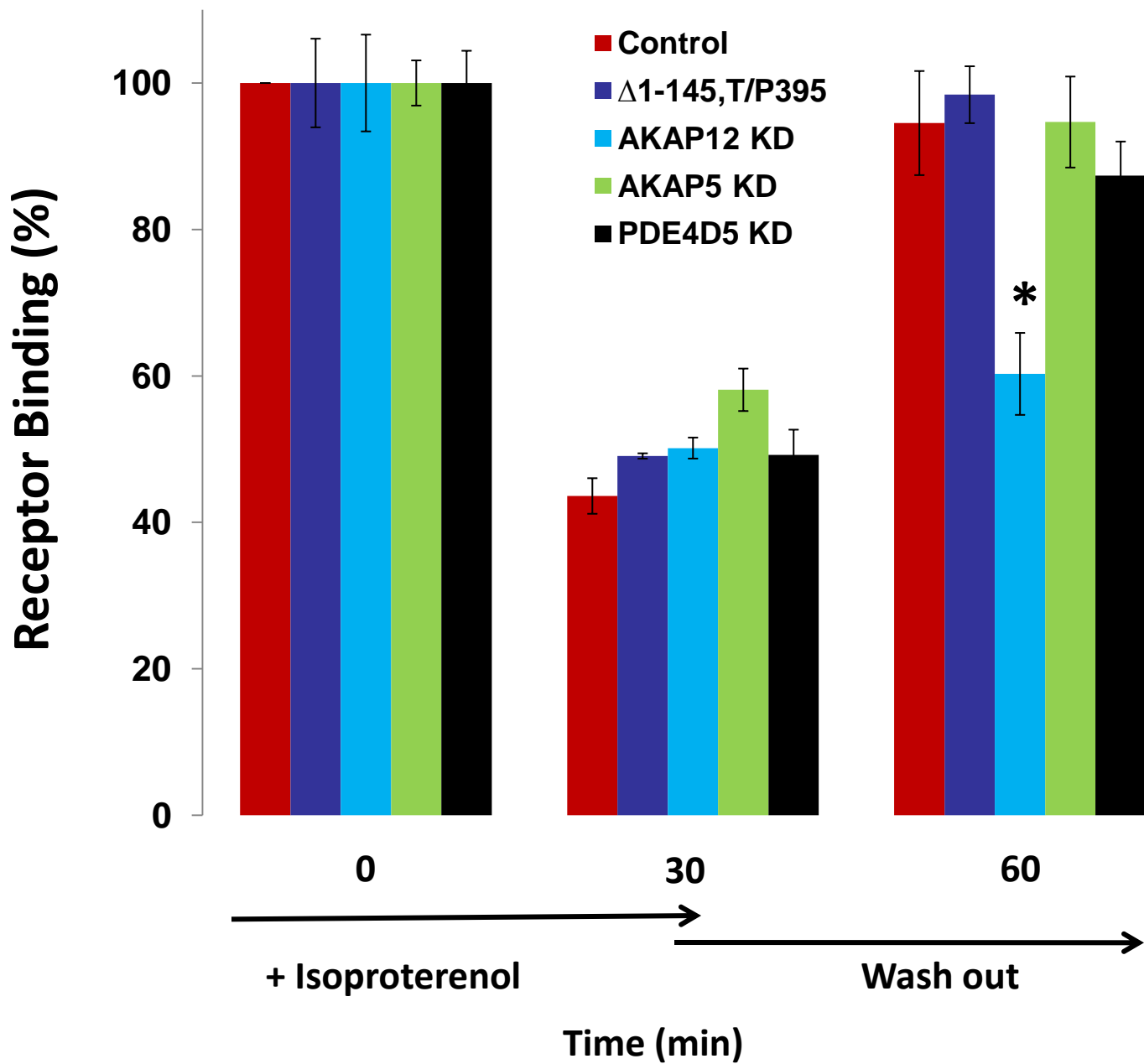

Supplement: Additional file 2 — Figure S2. Effects of targeted loss of AKAP5, AKAP12, and PDE4D5 on the resensitization/recycling of beta-adrenergic receptors. Experiments were performed with either wild-type A431 cells or cells stably transfected with an expression vector harboring the Δ1-145,T/P395 DIP of AKAP5. Cells were treated with siRNAs to knock-down (KD) AKAP12, AKAP5 or PDE4D5. These cells, deficient in AKAP5/12 or PDE4D5 then were treated with 10 μM ISO for 30 min to provoke full beta-adrenergic receptor desensitization and internalization (by 30 min). Cells then were washed free of agonist and incubated for additional 60 min to permit recovery. AKAP-mediated receptor resensitization/recycling, as measured directly using a membrane-impermanent radioligand that binds to only the complement of cell surface receptors (i.e., those receptors that have resensitized/recycled back to the cell membrane) was assayed to ascertain the impact of the expression of Δ1-145,T/P395 DIP of AKAP5 versus the loss of AKAP5, AKAP12, or PDE4D5. Cells not treated with ISO provided the control. The data shown are mean values plus/minus s.e.m. derived from at least three independent experiments. *, denotes significance of p < 0.05 from control cells at each time/condition. [file 1750-2187-7-4-S2.pdf]
